# Diabetic pulmonary microangiopathy and its association with glycated haemoglobin and other diabetic complications

**DOI:** 10.6026/973206300212186

**Published:** 2025-07-31

**Authors:** Lokesh M., Srinivasa S.V., Mani Mohan Reddy

**Affiliations:** 1 Department of General Medicine, Sri Devraj URS Academy of Higher Education And Research, Kolar, Karnataka, India

**Keywords:** Type 2 diabetes mellitus, pulmonary function tests, spirometry, glycated hemoglobin (HbA1C), albuminuria, diabetic retinopathy, diabetic nephropathy, restrictive lung disease

## Abstract

Diabetes mellitus is a systemic disease now recognized to affect the lungs through microangiopathy. This cross-sectional study assessed pulmonary function via spirometry in 132 diabetics and 132 controls. Diabetics showed significantly reduced FVC, FEV1 and PEFR, consistent with a restrictive pattern. Poor glycemic control correlated strongly with reduced lung function, while disease duration did not. Spirometry findings also correlated with albuminuria and retinopathy, supporting early screening utility.

## Background:

Diabetes mellitus (DM), especially Type 2 Diabetes Mellitus (T2DM), is one of the most common chronic non-communicable illnesses in the world, with its incidence and burden rising very fast in India [1]. DM is a multifactorial metabolic disorder that is defined by prolonged hyperglycemia due to insulin resistance and/or insufficient insulin secretion [2]. Chronic exposure to hyperglycemia causes diffuse damage to multiple organ systems, and complications have classically been divided into microvascular (retinopathy, nephropathy, neuropathy) and macrovascular (coronary artery disease, cerebrovascular accident, peripheral vascular disease) forms [3]. Complications are well-described and frequently tracked as components of diabetic management guidelines [4]. Yet, the effect of diabetes on lung function has not received due attention with increasing evidence accruing to the idea that diabetic microangiopathy has the lungs as a target organ. Lung is a very vascular organ with dense capillary bed, and its structural and functional integrity largely rests on microvascular integrity [5, 6]. Chronic hyperglycemia can provoke biochemical and structural alterations in pulmonary microvasculature and connective tissue, with the consequences of impaired gas exchange, stiffening of the lung parenchyma and reduced lung volumes-features of restrictive lung disease [7]. A number of population-based studies have indicated lowered values in pulmonary function tests like Forced Vital Capacity (FVC), Forced Expiratory Volume in one second (FEV_1_) and Peak Expiratory Flow Rate (PEFR) in diabetic patients, frequently even without overt respiratory symptoms [8]. These alterations have been attributed to non-enzymatic glycosylation of lung proteins, oxidative stress, alveolar epithelial basement membrane thickening, and augmented collagen deposition, all being diabetic microangiopathy pathophysiologic hallmarks [9]. In addition, severity of impairment of pulmonary function might be affected by glycemic control as measured through glycated hemoglobin (HbA1C) and potentially the duration of diabetes [10].

Certain research has suggested that suboptimal glycemic control worsens subclinical lung disease, whereas others have sought to correlate diabetic complications like nephropathy, retinopathy and neuropathy with decreasing lung function, suggesting an underlying microvascular etiology [11, 12]. As exciting as this new evidence is, routine pulmonary function testing is not yet part of the standard care for diabetes. Identification of pulmonary impairment in diabetics at an early phase is imperative for early intervention and avoidance of respiratory compromise [13]. Spirometry is a low-technology, non-invasive and inexpensive means of detecting alterations in lung function and can be a valuable adjunct in the monitoring of diabetic complications [14]. Therefore, it is of interest to assess pulmonary function in diabetics compared to non-diabetics and examine its association with glycemic control and microvascular complications.

## Materials and Methods:

This cross-sectional comparative study was performed in the Department of General Medicine at Sri Devraj Urs Medical College and Hospital, a constituent institution of Sri Devraj Urs Academy of Higher Education and Research (SDUAHER), Kolar, Karnataka, India. The study was conducted during a specified duration between 2020 and 2022 after getting clearance from the Institutional Ethics Committee. Informed consent was taken from all the participants before enrollment. 264 volunteers aged 18-70 years were enrolled in the study and grouped into two equal sets of 132 each: 132 patients with documented Type 2 Diabetes Mellitus and 132 age- and sex-matched healthy non-diabetic controls. The diagnosis of diabetes was made according to the American Diabetes Association (ADA) criteria, which are fasting plasma glucose ≥126 mg/dL, postprandial glucose ≥200 mg/dL, or HbA1C ≥6.5%. Inclusion criteria in the diabetic group were patients with Type 2 Diabetes Mellitus of any duration and who were clinically stable with no acute complications. The control group was constituted of non-diabetic patients with no chronic history of disease, age- and sex-matched. Exclusionary criteria for both groups were patients with a history of chronic respiratory illnesses like asthma, chronic obstructive pulmonary disease (COPD), pulmonary tuberculosis, and interstitial lung disease.

Patients with cardiovascular disease, active smokers, recent upper or lower respiratory infections, and patients with known neuromuscular conditions or recent thoracic/abdominal surgery were also excluded to exclude confounding factors on pulmonary function. Pulmonary function was measured with standardized spirometry. Recorded parameters included Forced Vital Capacity (FVC), Forced Expiratory Volume in one second (FEV_1_), the FEV_1_/FVC ratio, and Peak Expiratory Flow Rate (PEFR). All measurements were done with calibrated spirometers, with procedures performed by trained staff following American Thoracic Society/European Respiratory Society (ATS/ERS) guidelines. All participants executed at least three satisfactory maneuvers, and the best of those three was used. In diabetic patients, further testing consisted of measurement of glycated hemoglobin (HbA1C) levels with a standardized immunoturbidimetric assay. Microvascular complications were assessed using the following clinical measures: nephropathy was measured by an estimate of the urine albumin-creatinine ratio (UACR), where values >30 mg/g were taken as positive for microalbuminuria; retinopathy was detected by direct ophthalmoscopy or fundus photography and graded as non-proliferative or proliferative diabetic retinopathy; and neuropathy was assessed with monofilament examination and clinical evaluation of pain, vibration, and temperature.

## Results:

A total of 264 participants were included in this cross-sectional study, comprising 132 individuals with Type 2 Diabetes Mellitus and 132 age and sex-matched non-diabetic controls. Spirometric parameters were compared between groups, and correlations were explored between lung function and glycemic control, duration of diabetes, and the presence of microvascular complications.

The research involved 264 subjects who were equally distributed between non-diabetic and diabetic groups with similar age and gender distribution shown in Table 1 (see PDF). Diabetic patients had much lower pulmonary function values, mainly FVC, FEV1, and PEFR, than controls, ascertaining a restrictive pattern highlighted in Table 2 (see PDF). Stratified by glycemic control, the patients with HbA1C ≥7% had significantly lower spirometric indices compared to those who had better glycemic control, which showed a very good inverse correlation between HbA1C levels and lung function as compared in Table 3 (see PDF). Even though there was a trend towards declining FVC, FEV1, and PEFR with increasing duration of diabetes, this was not statistically significant shown in Table 4 (see PDF). A relevant correlation was noted between pulmonary impairment and diabetic nephropathy, with patients with microalbuminuria having decreased spirometry values than those with normal UACR highlighted in Table 5 (see PDF). Diabetic retinopathy was also related to noticeably diminished lung function parameters, particularly FVC and FEV1 depicted in Table 6 (see PDF). There was no statistically significant correlation noted between spirometry values and diabetic neuropathy highlighted in Table 7 (see PDF). The majority of diabetics exhibited a restrictive lung pattern, with 58.3% demonstrating such changes, and merely 9.8% and obstructive defect shown in Table 8 (see PDF). Pearson's correlation also established a moderate inverse correlation between HbA1C and pulmonary function parameters with PEFR correlating the most shown in Table 9 (see PDF). Substantially more diabetics exhibited abnormal spirometry patterns than non-diabetics (68.2% vs. 30.3%), as indicated in Table 10 (see PDF). Among both male and female, diabetics consistently had lower pulmonary lung function than non-diabetics as compared in Table 11 (see PDF). Finally, no significant relationship was identified between BMI and pulmonary parameters in the diabetic group as shown in Table 12 (see PDF).

## Discussion:

This cross-sectional study assessed pulmonary function in Type 2 Diabetes Mellitus patients compared to non-diabetic controls and its correlation with glycemic control and known microvascular complications [15]. The results clearly showed diabetic patients to have a significant impairment of vital spirometry parameters such as FVC, FEV_1_ and PEFR. These were indicative of a largely restrictive pulmonary pattern, as per the theory of diabetic pulmonary microangiopathy [16]. The lungs, although previously underappreciated as a target organ in diabetes, are structurally comparable to other microvascular-dense organs like the kidneys and retina [17]. This study confirms increasing literature that chronic hyperglycemia impacts the pulmonary microvasculature potentially via mechanisms such as thickening of basement membranes, oxidative stress and non-enzymatic glycation of structural proteins [18, 19]. As evidenced in our findings, the vast majority of the diabetic population-over two-third had impaired pulmonary function, restrictive defects being most prevalent [20]. One notable observation was the reversely related association between HbA1C and lung function indices. All patients with HbA1C ≥7% had lower FVC, FEV_1_, and PEFR compared to patients with improved glycemic control [21]. This supports previous findings that long-term poor glycemic control is a cause of pulmonary dysfunction. Correlation analysis also showed a moderate but significant negatively related correlation between levels of HbA1C and spirometric values, especially PEFR [22]. Even though no statistically significant correlation was observed between diabetes duration and pulmonary function in the study, a trend toward declining pulmonary function with increasing disease duration was noted [23]. This indicates that the cumulative, yet variable, burden of glycemia over time might affect pulmonary function in a similar manner, which might not always be apparent in cross-sectional imaging snapshots [24]. Both the occurrence of diabetic nephropathy and retinopathy were significantly correlated with decreased values of spirometry, pointing to the common microangiopathic etiology [25]. These results are consistent with earlier research that has suggested that pulmonary alterations can occur concomitantly with nephropathic and retinal involvement [26]. Conversely, no statistically significant correlation between pulmonary function and diabetic neuropathy was noted, implying different pathophysiologic processes [27, 28].

Sex-stratified analysis verified that diabetic men and women both had lower pulmonary parameters than their non-diabetic counterparts, even though the magnitude of depression did differ somewhat with gender [29]. BMI was not found to have a significant influence on pulmonary function in the diabetic group, suggesting that changes seen are more diabetes-related than obesity-related per se [30]. This research very much attests to the status of pulmonary dysfunction as a significant, yet silent, complication of Type 2 Diabetes Mellitus. Regular spirometric screening, particularly in cases with poor glycemic control or microvascular disease, could help facilitate early detection and management of respiratory impairment.

## Conclusion:

Type 2 diabetes is associated with significant restrictive impairment in pulmonary function. This impairment correlates strongly with poor glycemic control and microvascular complications. Routine spirometry may aid in early detection and better long-term respiratory outcomes in diabetics.
